# The Effects of Prophylactic Dexmedetomidine Administration on General Anesthesia Recovery Quality in Healthy Dogs Anesthetized With Sevoflurane and a Fentanyl Constant Rate Infusion Undergoing Elective Orthopedic Procedures

**DOI:** 10.3389/fvets.2021.722038

**Published:** 2021-09-28

**Authors:** Sarah K. Jarosinski, Bradley T. Simon, Courtney L. Baetge, Stephen Parry, Joaquin Araos

**Affiliations:** ^1^Department of Small Animal Clinical Sciences, College of Veterinary Medicine and Biomedical Sciences, Texas A&M University, College Station, TX, United States; ^2^Cornell Statistical Consulting Unit, Cornell University, Ithaca, NY, United States; ^3^Department of Clinical Sciences, Cornell University College of Veterinary Medicine, Ithaca, NY, United States

**Keywords:** anesthesia, constant rate infusion, dexmedetomidine, dog, dysphoria, fentanyl, recovery, sevoflurane

## Abstract

To determine the effects of a dexmedetomidine slow bolus, administered prior to extubation, on recovery from sevoflurane-anesthesia and a fentanyl continuous rate infusion (CRI) in dogs undergoing orthopedic surgical procedures. Sixty-two client-owned, healthy dogs weighing 27.4 ± 11 kg undergoing elective orthopedic procedures were premedicated with: 0.1 mg/kg hydromorphone intramuscular, 0.05 mg/kg hydromorphone intravenously (IV) or 5 mcg/kg fentanyl IV. Following premedication, dogs were induced with propofol, administered locoregional anesthesia and maintained with sevoflurane and a fentanyl CRI (5–10 mcg/kg^/^hr). Dogs were randomly assigned to one of two treatment groups: 0.5 mcg/kg dexmedetomidine (DEX) or 0.5 ml/kg saline (SAL). Following surgery, patients were discontinued from the fentanyl CRI and administered DEX or SAL IV over 10 min. Following treatment, dogs were discontinued from sevoflurane and allowed to recover without interference. Recoveries were video recorded for 5 min following extubation and assessed by two blinded anesthesiologists using a visual analog scale (VAS; 0–10 cm) and a numerical rating scale (NRS; 1–10). Mean arterial pressure (MAP), heart rate (HR), pulse oximetry (SpO_2_), temperature, respiratory rate (RR), and end-tidal sevoflurane (EtSevo) and carbon dioxide (EtCO_2_) concentrations were recorded at specific time-points from induction to 5 min post-bolus administration and analyzed using linear mixed models. Fentanyl, propofol, and hydromorphone dose and the time to extubation were compared using an unpaired *t*-test. Differences in recovery scores between groups were evaluated with a Mann-Whitney test. Data reported as mean ± SD or median [interquartile range] when appropriate. A *p* < 0.05 was significant. There were no significant differences between groups in fentanyl, propofol, and hydromorphone dose, duration of anesthesia, intraoperative MAP, HR, RR, SpO_2_, temperature, EtCO_2_, EtSevo or anesthetic protocol. MAP was higher in DEX compared to SAL at 10 (104 ± 27 and 83 ± 23, respectively) and 15 (108 ± 28 and 86 ± 22, respectively) min after treatment. DEX had significantly lower VAS [0.88 (1.13)] and NRS [2.0 (1.5)] scores when compared to SAL [VAS = 1.56 (2.59); NRS = 2.5 (3.5)]. Time to extubation (min) was longer for DEX (19.7 ± 11) when compared to SAL (13.4 ± 10). Prophylactic dexmedetomidine improves recovery quality during the extubation period, but prolongs its duration, in sevoflurane-anesthetized healthy dogs administered fentanyl.

## Introduction

Post-anesthesia delirium and excitation (i.e. dysphoria) have been reported in 20–25% of dogs undergoing surgical procedures following the administration of fentanyl constant rate infusions (CRI) and volatile anesthetics (1). Associated behaviors involve the acute onset of restlessness, agitation, hyperactivity, thrashing, non-purposeful movement and inconsolability (2). Excitation and/or dysphoria during the recovery period can result in patient and personnel harm and stress, destruction of anesthesia equipment, removal of intravenous (IV) access, and resistance to handling or restraint (3, 4). These adverse effects are costly in terms of resources and may increase length of hospital stays, morbidity, and mortality (5).

The exact pathophysiological mechanisms that result in post-anesthesia delirium or dysphoria remain unknown (4). It is hypothesized that volatile anesthetics (e.g. sevoflurane) result in a disparity in the rate of anesthetic elimination from areas of the brain associated with audition and locomotion (early recovery) and cognition function (late recovery) (3, 5). A delay in the recovery of the subcortical thalamoregulatory systems following general anesthesia, hampering the integration of cortical information, may also contribute to post-anesthetic confusion and agitation (6). Little research has evaluated this phenomena in veterinary medicine and much of what is discussed pertains to drug-induced post-anesthetic dysphoria associated with opioid analgesics (1).

The management of post-anesthesia dysphoria or excitation in dogs often includes the administration of an IV sedative. Alpha_2_ adrenoreceptor agonists (e.g. dexmedetomidine or medetomidine) have been incorporated into veterinary anesthesia premedication and intraoperative protocols for decades, in part, because of their favorable sedative and adjunctive analgesic effects that may last well into the recovery period (7). However, the high premedication doses required to achieve such effects on the recovery period may result in significant intraoperative cardiovascular compromise (7). Therefore, timing of administration has been recently focused to the period following extubation to improve or smooth the transition from general anesthesia to recovery (8, 9). This technique may minimize intraoperative cardiovascular complications, however, administering dexmedetomidine or medetomidine following extubation may not prevent these unwanted excitatory behaviors. Furthermore, administration techniques (e.g. dosing and speed of delivery) previously evaluated in dogs are still similar to those administered as premedication (8, 9). In human patients, dexmedetomidine (0.5 or 1 mcg/kg) administered over 10 min immediately prior to extubation reduced the incidence of post-anesthesia delirium or dysphoria and improved recovery quality following general anesthesia, without prolonging the time to extubation (10). To the authors' knowledge, studies evaluating the efficacy of administering a prophylactic dose of dexmedetomidine as a slow bolus immediately prior to recovery to reduce the incidence of post-anesthesia excitation or dysphoria in dogs anesthetized with sevoflurane and a fentanyl CRI has not been reported.

The primary objective was to evaluate and compare recovery quality following the administration of a low-dose dexmedetomidine or saline IV slow bolus immediately prior to extubation in sevoflurane anesthetized healthy dogs. The primary hypothesis was that dexmedetomidine would improve recovery quality in dogs when compared saline. The secondary objective was to evaluate recovery duration under the same conditions as previously stated. The secondary hypothesis was that the duration of recovery would be similar in dogs administered IV dexmedetomidine or saline.

## Materials and Methods

This study was approved by the Texas A&M University's College of Veterinary Medicine and Biomedical Sciences Institute's Animal Care and Use Committee and was approved (IACUC 2018-0045).

### Animals

Sixty-two client owned, young adult or adult dogs, presenting to Texas A&M Veterinary Medical Teaching Hospital (VMTH) for elective or non-emergent orthopedic surgery and assigned an American Society of Anesthesiologists Physical Status (ASA PS) of I or II were recruited for the study. ASA PS was based on history, physical examination, complete blood count and serum biochemistry panel. Dogs were excluded from the study if they had central nervous system disease, newly diagnosed heart murmur on presentation to the VMTH, cardiomegaly as diagnosed on a lateral thoracic radiograph or echocardiogram, valvular disease of stage B2 or greater [American College of Veterinary Internal Medicine “stages of heart disease and failure in dogs” classification (11)], brachycephalic conformation, or aggressive and/or significant nervous behaviors such as growling, piloerection, uncooperative behavior during minimal restraint and snapping. Dogs were also excluded if administered acepromazine, an α_2_ adrenoreceptor agonist, ketamine, or a benzodiazepine within 12 h prior to anesthesia and surgery associated with this present study.

### Anesthesia Protocol and Intraoperative Period

Dogs were fasted, but water was provided ad libitum, for a minimum of 8 h prior to premedication and anesthesia. All dogs were premedicated with one of the following: 0.1 mg/kg hydromorphone (Hydromorphone HCl 2mg/ml,West-Ward, Eatontown, NJ, USA) intramuscular (IM), 0.05 mg/kg hydromorphone IV or 5 mcg/kg fentanyl (Fentanyl Citrate 50 mcg/mL, Hospira, Lake Forest, IL, USA). Patients received an IV premedication if an IV cannula was preplaced prior to presentation to the anesthesia service (*n* = 7). Patients who received an IM premedication (*n* = 55) had a small area of hair clipped and the adjacent skin aseptically prepared for an appropriately sized IV cannula to be placed and secured in either a cephalic or lateral saphenous vein 15 min following their premedication injection. Propofol (Propofol 10mg/mL, Abbott, Chicago, IL, USA) was administered through the IV cannula slowly and to effect (e.g. medial ventral eye rotation, lack of palpebral reflex, and no swallowing or gagging reflex during laryngoscope placement) until endotracheal intubation was achievable with an appropriately sized cuffed endotracheal tube. Endotracheal tubes were attached to a rebreathing circuit and dogs were administered sevoflurane (Sevoflurane, Zoetis, Parsippamy-Troy Hills, NJ, USA) mixed in a carrier gas of 100% oxygen. Heart rate (HR), end-tidal CO_2_ (EtCO_2_), respiratory rate (RR), end-tidal sevoflurane concentration (EtSevo), rectal or esophageal temperature, arterial oxygen saturation (SpO_2_), and oscillometric blood pressure using an appropriately sized blood pressure cuff (e.g. 30-40% the circumference of the limb) were recorded every 5 min using a multiparameter anesthesia monitor (Mindray, Mahwah, NJ, USA). All dogs were placed on a mechanical ventilator (Hallowell EMC, Pittsfield, MA) and provided positive pressure ventilation at 8 to 12 breaths per min along with a peak inspiratory pressure of 10–20 cmH20 to maintain an EtCO_2_ between 35-45 mmHg. Following induction and placement of monitoring devices, all dogs except two, were administered a fentanyl IV bolus of 5 mcg/kg and then started on a fentanyl CRI using a syringe pump (B Braun Medical, Bethlehem, PA, USA), for inhalant sparing effects, and continued throughout surgery and into the recovery period. The two dogs that did not receive a fentanyl bolus at this time (one dog in each experimental group), received their fentanyl bolus as part of their premedication just prior to induction. Fentanyl CRI dose was started at 5 mcg/kg/hr and adjustments were determined by the attending nurse anesthetist or veterinary anesthesia resident and supervising anesthesiologist (Table 1). Fentanyl doses were increased or decreased at 1–2 mcg/kg/hr intervals based on the patient's requirements for nociceptive relief. Fentanyl dose was adjusted intraoperatively based on fluctuations in physiological parameters (e.g. ±20% change in heart rate and blood pressure) and anesthetic depth. Patients were then aseptically prepped for their scheduled surgical orthopedic procedure(s). During the aseptic preparation, the patient was administered pre-operative antibiotics [Cefazolin (Cefazolin sodium1g, Apotex Corps, Toronto, CA) 22 mg/kg IV] as is standard hospital protocol. Following the aseptic preparation and prior to surgery, all patients were administered an appropriate local regional technique based upon the location of the surgical procedure (Table 1). For example, pelvic limb procedures received a 0.5% bupivacaine (Bupivacaine HCl 0.5%, Hospira, Lake Forest, IL, USA) and morphine (Morphine 10 mg/ml, Hikma Pharmaceuticals, London, UK) (0.1 mg/kg) lumbosacral epidural or a sciatic and femoral nerve block with 0.5% bupivacaine (0.2 ml/kg), and/or a subcutaneous injection of liposomal encapsulated bupivacaine [Bupivacaine liposome injectable solution (Nocita) 13.3 mg/mL, Elanco, Greenfield, IN, USA] (0.4 ml/kg) at the incision site. Thoracic limb procedures were administered a radial ulnar musculocutaneous median nerve block or brachial plexus nerve block with 0.5% bupivacaine (0.2-0.4 ml/kg and 0.3 ml/kg, respectively), and/or a subcutaneous injection of liposomal encapsulated bupivacaine (0.4 ml/kg) at the incision site. Anesthetic depth was monitored throughout and deemed appropriate as loss of palpebral reflex, ventromedial rotation of the eye and lack of jaw tone and purposeful response to surgical stimulation. All patients enrolled in the study were monitored by a highly trained nurse anesthetist or American College of Veterinary Anesthesia and Analgesia trained resident who was blinded to the assigned treatment group.

**Table 1:** Comparison of sex, surgical procedure type, age, weight, total dose of hydromorphone and fentanyl, and duration of sevoflurane anesthesia in dogs undergoing an elective or non-emergent orthopedic procedure.

**Variable**	**SAL**	**DEX**	***P*-value**
Male:Female (*n*)	12:19	18:13	0.204
TPLO:Other orthopedic surgical procedures (*n*)	21:10	17:14	0.435
Age (years)	4.8 ± 2.9	4.6 ± 2.8	0.309
Weight (kg)	27.1 ± 11.9	27.6 ± 10.8	0.547
Hydromorphone dose (mg/kg)	0.1 ± 0.03	0.1 ± 0.02	0.900
Propofol dose (mg/kg)	3.7 ± 1.3	4.1 ± 1.4	0.205
Total CRI fentanyl amount administered perioperatively (mcg)	478.4 ± 339	544.1 ± 322	0.434
Fentanyl CRI dose (mcg/kg/hr)	5.96 ± 1.8	5.80 ± 2.0	0.745
Duration of anesthesia (h)	3.0 ± 1.1	3.5 ± 1.0	0.079
**Local regional technique performed (** ***n*** **)**			
Lumbosacral epidural	23	23	0.672
Sciatic/femoral nerve block	1	0	
RUMM nerve block	1	3	
Brachial plexus block	1	0	
SC incisional infiltrative block and one of the above blocks	24	26	
SC incisional infiltrative block alone	5	4	

*Dogs were administered either an intravenous slow bolus, over 10 min, of 0.9% sterile saline (10 mL, SAL; n = 31) or dexmedetomidine (0.5 mcg/kg diluted with 0.9% sterile saline to 10 mL, DEX; n = 31) following completion of their assigned procedure, but immediately prior to discontinuation of the inhalant anesthetic. Age, weight, hydromorphone, propofol, and fentanyl CRI dose, total fentanyl amount administered, and duration of anesthesia are represented as mean ± standard deviation*.

*TPLO, Tibial plateau leveling osteotomy; CRI, Constant rate infusion; RUMM, Radial, ulnar, and musculocutaneous median; SC, subcutaneous*.

### Post-operative Experimental Treatment Period and Recovery

Upon completion of the scheduled orthopedic surgical procedure(s), the fentanyl CRI was set to and maintained at 5 mcg/kg/hr and dogs were discontinued from the ventilator and transported to radiology for post-operative radiographs. Throughout this period, patients remained under general anesthesia with the same transportable anesthesia delivery system that was utilized during induction and surgery. Following radiographs, dogs were transported to the quiet recovery area for icing of the incision and bandaging on the transport gurney. During this time (i.e. icing and bandaging), the anesthetist maintained manual ventilation at a rate of 2–4 breaths per min until the dogs began spontaneously ventilating. The dogs were then randomly assigned using online software (www.randomizer.org) to 1 of 2 groups: dexmedetomidine (Dexmedetomidine HCl 0.5 mg/mL, Zoetis, Florham Park, NJ, USA) (0.5 mcg/kg IV; DEX) diluted to 10 mL with 0.9% sterile saline (Baxter International, Deerfield, IL, USA; Methadone HCl 10 mg/mL, Akorn, Lake Forest, IL, USA)or 0.9% sterile saline (10 mL IV; SAL). Fentanyl CRIs were discontinued upon starting DEX or SAL. DEX and SAL were both started as a slow IV bolus over 10 min using a syringe pump (B Braun Medical, Bethlehem, PA, USA), during the final stages of the bandage placement.

During the slow bolus, patients were maintained under sevoflurane-anesthesia until completion of DEX or SAL. Sevoflurane vaporizer dial settings immediately prior to DEX or SAL treatment administration were kept consistent and maintained throughout their administration. Anesthetists were instructed to not alter the sevoflurane setting on the vaporizer during this recovery period until after the slow bolus was complete. DEX and SAL dosing and timing of administration were based on unpublished pilot data and current practices performed by the investigators. To maintain the blinded integrity of the study, DEX or SAL dilutions were made by the principal investigator (SJ) in non-identifying syringes prior to anesthesia recovery and hand-delivered to the patient's anesthetist. Anesthetists administering the treatment slow bolus were blinded to drug administration to avoid bias with regard to pharmaceutical management in the emergence period. HR, mean arterial pressure (MAP), respiratory rate (RR), electrocardiogram (ECG) and SpO_2_ were recorded immediately prior to starting DEX or SAL (time 0; TO) and at 5, 10, and 15 (i.e., 5 min following) completion of the infusion) min after starting the bolus. After the administration of DEX or SAL (i.e., immediately after 10 min following treatment administration recordings), sevoflurane anesthesia was discontinued. Patients remained connected to the anesthesia machine and were administered 100% oxygen for 5 min following the discontinuation of sevoflurane (i.e., up to and including the 15-min post-treatment administration recording). Afterward, they were disconnected from the anesthesia machine and allowed to ventilate on room air and recover from general anesthesia on a padded transport gurney in a quiet environment without auditory or physical stimulation. Anesthetists were instructed to administer flow-by oxygen if necessary to maintain and SpO_2_ measurement above 96% during the post-anesthetic period. Finally, anesthetists were allowed to administer IV rescue sedation (dexmedetomidine (0.5–1 mcg/kg) or acepromazine (0.005–0.01 mg/kg) to any animal following extubation that exhibited immediate signs of severe thrashing, extensive vocalizing, or aggressive tendencies that could impose harm on themselves or the personnel. Immediately, following the 15-min treatment assessment period, postoperative opioid analgesics (e.g. methadone; Dexmedetomidine HCl 0.5 mg/mL, Zoetis, Florham Park, NJ, USA [0.2 mg/kg IV] or hydromorphone [0.05 mg/kg IV] pending clinician preference and drug availability) were administered if a patient scored higher than a one out of four (e.g., score two or greater) on the Colorado Acute Pain Scale utilized by the hospital's post-anesthesia care unit.

Recovery duration was recorded (time from discontinuation of inhalant anesthetic to extubation) for each patient. Extubation was determined if a prominent and persistent gag or swallowing reflex or chewing was noted by the blinded anesthetist. Recoveries were video recorded for later evaluation using the anesthetist's cell phone camera which was propped on a stand to allow complete visualization of the patient during the recovery period. Videos were trimmed using computer software to include from time of and including extubation to 5 min thereafter. All trimmed videos were collated, randomly assigned an order for evaluation, and distributed to two blinded, board-certified American College of Veterinary Anesthesia and Analgesia anesthesiologists (BS and CB) who scored the recoveries using a numerical rating (0–10; 0 = best recovery possible, 10 = worst recovery possible) and visual analog scales (0–100 mm) previously published elsewhere (9, 12). The incidence of dysphoria during recovery was determined using the averaged NRS score generated from both blinded anesthesiologists evaluating the recoveries. Dogs with an average NRS recovery score of five or greater out of 10 would later be classified as an “unacceptable recovery” and is extrapolated from a previous study (1). An injectable non-steroidal anti-inflammatory (e.g. carprofen 2.2 mg/kg SC) was administered by the surgery service when the patient was deemed normothermic and alert and following the 5-min recovery quality assessment period.

### Statistical Analysis

A priori power analysis suggested 62 total dogs (DEX [n=31]; SAL [n=31]) would be needed using an alpha probability value of 0.05 and beta power of 0.8 with an effect size of 0.75 in order to detect a difference of 1.5 in the NRS recovery score.

Normality of data distribution was evaluated with the Shapiro-Wilk test. Normally distributed data are reported as mean ± standard deviation and non-normally distributed data as median [interquartile range]. Differences in patient demographics, hydromorphone premedication and propofol dose, fentanyl CRI dose and total fentanyl administered perioperatively, local regional technique(s) performed, duration of anesthesia and duration of recovery between groups were compared with a two-tailed *t-*test or Fischer's exact test, depending on data distribution. Differences in specific intraoperative and treatment bolus physiologic and anesthetic variables were compared between groups analyzed with linear mixed models, with time and group and the interaction between time and group as fixed effects, and the dog as the random effect. Intraoperative variables recorded at 30-min intervals were used for analysis. *Post hoc* between groups at each time point during intraoperative and treatment bolus times were performed and corrected with Bonferroni for multiple comparisons. Intragroup differences for each variable during intraoperative or post-operative (i.e. the 15 min associated with the treatment bolus) study times were compared against its respective baseline and treatment time 0 (immediately prior to slow bolus treatment administration; T0), respectively, with the Dunnet's test. Interrater agreement was evaluated with intraclass correlation coefficient [ICC (95% CI)]. After confirming adequate ICC, the average of the 2 reviewers for each recovery score system was used for further statistical analysis. Differences in averaged visual analog and numerical rating scale recovery scores between groups were evaluated with a two-tailed Mann-Whitney test. Since there were no differences in potential confounding factors for quality of recovery between groups such as duration of anesthesia, total hydromorphone and total fentanyl doses, no further tests were performed. A *p* < 0.05 was considered statistically significant. Data was analyzed with JMP Pro 15 (SAS, USA) and MedCalc (MedCalc Software Ltd, Belgium).

## Results

All dogs enrolled successfully completed the study and were included for statistical evaluation. None of the dogs required additional intraoperative analgesics other than their locoregional technique and the fentanyl CRI. There were no significant differences between groups with respect to age, weight, sex, type of orthopedic procedure, hydromorphone and propofol dose, fentanyl CRI dose and total amount of fentanyl administered, local regional technique(s) performed, and duration of anesthesia (Table 1). Breeds represented included akita (n=1), American Pit Bull Terrier (n=2), American Staffordshire Terrier (*n* = 1), Australian Cattle Dog (*n* = 1), Australian Shepherd (*n* = 5), Basset Hound (*n* = 1), Bichon Frise (*n* = 1), Bloodhound (*n* = 1), Border Collie (*n* = 2), Boston Terrier (*n* = 1), Boxer (*n* = 3), Bull Mastiff (*n* = 1), Catahoula Hog Dog (*n* = 1), Doberman Pinscher (*n* = 1), German Shepherd (*n* = 3), Golden Retriever (*n* = 2), Great Dane (*n* = 1), Great Pyrenees (*n* = 1), Labrador Retriever (*n* = 11), Maltese (*n* = 1), Miniature Australian Shepherd (*n* = 1), Miniature Schnauzer (*n* = 1), mixed breed (*n* = 9), Nova Scotia Duck Tolling Retriever (*n* = 1), Old English Sheepdog (*n* = 1), Papillon (*n* = 1), Pit Bull (*n* = 1), Plott Hound (*n* = 1), Rottweiler (*n* = 2), Vizsla (*n* = 1), Whippet (*n* = 1), Yorkshire Terrier (*n* = 1).

### Intraoperative Variables

There were no differences between or within groups in SpO_2_, EtCO_2_, EtSevo, or temperature at any time point (Tables 2, 3). HR was significantly lower in SAL intraoperatively at 60 and 90 min when compared with baseline. HR was significantly lower in DEX when compared to SAL at intraoperative baseline.

**Table 2:** Mean ± standard deviation of intraoperative period heart rate, mean arterial pressure, peripheral capillary oxygen saturation, and end-tidal carbon dioxide pressure and sevoflurane concentration of dogs described in Table 1.

		**Time (min)**							
**Variable**	**Group**	**(Baseline)**	**30**	**60**	**90**	**120**	**150**	**180**	***P -* value**
HR (beats per min)	*SAL*	86 ± 26	72 ± 20	67 ± 16^†^	69 ± 16^†^	71 ± 18	73 ± 15	75 ± 20	0.078
	*DEX*	74 ± 28^*^	67 ± 22	65 ± 22	63 ± 19	68 ± 20	65 ± 16	68 ± 19	
MAP (mmHg)	*SAL*	85 ± 19	76 ± 11	73 ± 13	76 ± 13	76 ± 14	78 ± 18	79 ± 19	0.026
	*DEX*	79 ± 17	75 ± 15	77 ± 15	74 ± 12	80 ± 16	76 ± 14	81 ± 13	
SpO_2_ (%)	*SAL*	98 ± 2	98 ± 2	99 ± 1	99 ± 1	99 ± 1	99 ± 2	99 ± 2	0.096
	*DEX*	98 ± 1	98 ± 2	98 ± 2	98 ± 2	99 ± 2	99 ± 1	99 ± 1	
EtCO_2_ (mmHg)	*SAL*	NR	38 ± 4	39 ± 3	39 ± 4	40 ± 3	38 ± 4	40 ± 3	0.289
	*DEX*	NR	39 ± 3	40 ± 4	41 ± 3	41 ± 5	38 ± 9	41 ± 6	
EtSevo (%)	*SAL*	NR	2.0 ± 0.3	2.0 ± 0.4	1.8 ± 0.5	1.9 ± 0.8	1.9 ± 0.6	2.1 ± 0.5	0.429
	*DEX*	NR	2.1 ± 0.4	2.0 ± 0.4	2.0 ± 0.3	2.1 ± 0.3	2.2 ± 0.2	2.2 ± 0.2	
Temperature (°F)	*SAL*	97.7 ± 2.3	96.7 ± 1.7	96.8 ± 1.7	97.1 ± 1.6	97.6 ± 1.6	98.1 ± 1.6	98.3 ± 1.8	0.706
	*DEX*	97.5 ± 2.2	96.4 ± 1.8	96.3 ± 2.1	96.9 ± 1.9	97.4 ± 1.9	97.6 ± 1.9	98.3 ± 2.2	

*Parameters were measured every 5 min but recorded every 30 min for analysis. Parameters were recorded from immediately prior to induction of anesthesia (baseline) to 180 min following induction of anesthesia (Time = 180), but prior to starting SAL or DEX*.

*HR, heart rate; MAP, mean arterial pressure; SpO2, peripheral capillary oxygen saturation; EtCO_2_, end-tidal carbon dioxide pressure; EtSevo, end-tidal sevoflurane concentration; NR, not recorded*.

* *p < 0.05 compared with group SAL in the same time period*.

† *p < 0.05 compared with time 0 within group*.

**Table 3:** Mean ± standard deviation of post-operative treatment period heart rate, mean arterial blood pressure, respiratory rate, and peripheral capillary oxygen saturation of sevoflurane anesthetized dogs randomly assigned to receive dexmedetomidine (0.5 mcg/kg IV; DEX) diluted to 10 mL with 0.9% sterile saline or 0.9% sterile saline (10 mL IV; SAL) administered as a slow IV bolus over 10 min.

		**Time (min)**				
**Variable**	**Group**	**0 (T0)**	**5**	**10**	**15**	***P*- value**
HR (beats per minute)	*SAL*	74 ± 19	75 ± 21	75 ± 21	76 ± 24	0.04
	*DEX*	72 ± 18	68 ± 17	65 ± 14	65 ± 16^*^	
MAP (mmHg)	*SAL*	78 ± 12	82 ± 20	83 ± 23	86 ± 22	0.026
	*DEX*	80 ± 16	94 ± 22^*^^†^	104 ± 27^*^^†^	108 ± 28^*^^†^	
SpO_2_ (%)	*SAL*	99 ± 2	99 ± 1	99 ± 2	99 ± 2	0.096
	*DEX*	99 ± 1	99 ± 2	99 ± 1	99 ± 1	
RR (breath per minute)	*SAL*	12 ± 7	13 ± 8	13 ± 8	15 ± 12^†^	0.330
	*DEX*	14 ± 6	15 ± 8	15 ± 8	14 ± 8	

*Parameters were measured every 5 min from immediately prior to starting DEX or SAL (Time = 0; T0) to 5 min after DEX or SAL was complete (Time = 15)*.

*HR, heart rate; MAP, mean arterial pressure; SpO_2_, peripheral capillary oxygen saturation; RR, respiratory rate*.

* *p < 0.05 compared with group SAL in the same time period*.

† *p < 0.05 compared with time 0 within group*.

### Post-operative Treatment Variables

HR was significantly lower at 15 min for DEX when compared to SAL. In group DEX, MAP was significantly higher at 5, 10, and 15 min when compared with T0 min. Group DEX had a higher MAP compared to SAL at 5, 10, and 15 min during the treatment period. There were no ECG abnormalities noted at any time point for DEX or SAL. In group SAL, RR was significantly higher at 15 min post-treatment when compared with T0 min within the same treatment (15 ± 12 and 12 ± 7, respectively). However, RR was not different between SAL and DEX at any time point.

### Recovery Variables

**Figure 1:**
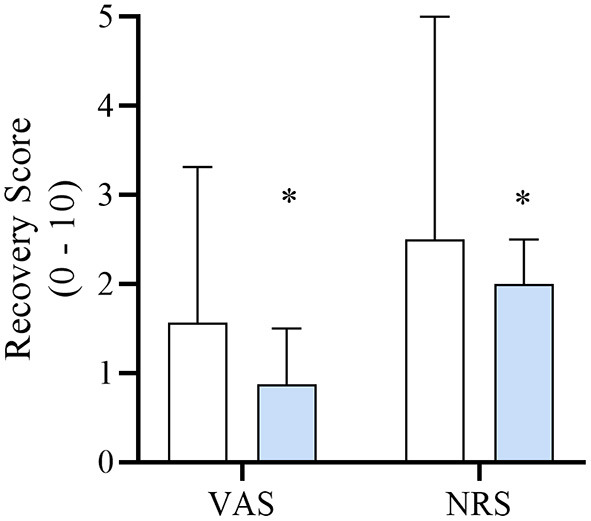
Median [interquartile range] numerical rating scale (NRS; 0–10) and visual analog scale (VAS; 0–10 cm) recovery scores of dogs in each treatment group DEX (blue) or SAL (white) defined in Table 1. *Significantly lower when compared to SAL.

Recovery duration was significantly longer for DEX when compared to SAL (19.7 ± 11 and 13.4 ± 10 min, respectively, *p* = 0.03). Intra-class correlation was excellent for VAS [0.93 (0.89–0.96)] and for NRS [0.91 (0.85–0.95)]. DEX had significantly lower VAS [0.88 (1.13); *p* = 0.026] and NRS [2.0 (1.5); *p* = 0.041] recovery scores when compared to SAL [VAS = 1.56 (2.59); NRS = 2.5 (3.5)] (Figure 1). Overall, 9/62 (15%) dogs had an unacceptable recovery. Specifically, SAL, and DEX group had 6/30 (20%) and 3/32 (9%) unacceptable recoveries, respectively. Two patients in the DEX group required rescue analgesia (hydromorphone [*n* = 1] and methadone [*n* = 1]) following extubation and assessment of the recovery quality. No dogs in the SAL group required rescue analgesia. Following extubation, both dogs that required rescue analgesia exhibited no behaviors associated with dysphoria. Averaged VAS and NRS recovery scores for each dog were 0.125 cm and 0, and 1cm and 2, respectively.

## Discussion

The administration of a low-dose dexmedetomidine slow bolus immediately before discontinuing sevoflurane anesthesia improved the quality of recovery in healthy dogs receiving a fentanyl CRI while undergoing an elective or non-emergent orthopedic surgical procedure. In addition, dogs who received dexmedetomidine had heart rates lower and arterial blood pressure higher when compared to dogs administered saline. However, these values remained within clinically acceptable ranges. Finally, the administration of dexmedetomidine resulted in a longer recovery time, however the 6-min difference between groups may be considered clinically insignificant.

Dysphoria has been described in humans as the feeling of unpleasantness (13). There are many applications of this word to various organ systems and emotions, and the definition is both broad and vague. The mechanism of dysphoria is poorly understood in veterinary medicine. Opioids interact with multiple receptors, but the μ-, κ-, and δ-opioid receptors are of the most interest. In humans, κ-opioid agonist drugs are more likely to cause dysphoria after administration (1). However, it is unclear if the same is true across species. Genetics may also be a factor in an individual's response to an opioid. Humans who have variations in the multidrug resistance-1(MDR-1) gene report a higher incidence of hallucinations (14). Though a few of the dogs enrolled in this study were northern breeds or sight hounds, breeds known for having a higher likelihood of MDR-1 gene variation, distribution of these breeds between the DEX and SAL groups were similar.

Post-anesthetic dysphoria has been studied in adults and children with incidence rates of 4.7% to 22.2 and 10 to 80%, respectively (15, 16). The administration of opioids during inhalant anesthesia has been previously reported to increase the risk of post-anesthetic dysphoria in animals (1, 17, 18). Specifically, fentanyl, a μ-opioid receptor agonist, has been reported to induce clinical signs associated with dysphoria in humans and dogs (1, 19). The prevalence of post-anesthesia dysphoria associated with fentanyl administration in dogs can be up to 34% (1). Incidence rates of dysphoria associated with morphine administration are similar to those observed with fentanyl in dogs (18, 20). Despite these previous studies reporting incidences it is still not well understood why opioids contribute to post-anesthetic dysphoria in dogs (17). Interestingly, dysphoria may be less commonly observed in pain-free dogs administered a fentanyl CRI of short duration (i.e. <60 min) (21).

Techniques used to diagnose dysphoria (i.e. confirmation of adequate pain control followed by an increase in intensity of dysphoric behaviors followed by additional [rescue] opioid administration) were not incorporated into this study design. Therefore, it is difficult to determine if the post-anesthesia behaviors observed were that of dysphoria or of from another cause (i.e. agitation, excitement, pain, stress). Instead, NRS results were analyzed to determine the incidence of unacceptable recoveries in patients that resemble similar behavioral characteristics as observed during post-anesthesia dysphoria (1). The percentage of SAL group dogs experiencing unacceptable recoveries in the present study (20%) is consistent with a previous publication evaluating the prevalence of dysphoria after fentanyl in dogs undergoing stifle surgery (1). In the latter study, the prevalence of post-anesthesia dysphoria in dogs administered a fentanyl CRI at 2, 5 or 10 mcg/kg/h was 20%, 34% or 17%, respectively. The administration of dexmedetomidine as described in the present study resulted in a lower incidence of post-anesthesia dysphoric-like behaviors than compared to previous studies in which anesthetized dogs were administered opioids without dexmedetomidine (1, 18, 20). However, one should interpret these comparisons with caution due to the differences in dosing regimens, surgical procedures, opioids administered, and population demographics. Despite these differences, veterinarians should consider this dosing regimen in healthy dogs where excitation or dysphoria may be likely following anesthesia.

The management of a poor anesthetic recovery following general anesthesia routinely involves the administration of a sedative (e.g. dexmedetomidine). In adult and pediatric human patients, dexmedetomidine has been reported to reduce dysphoria (10, 22–24). In those studies, various protocols have been studied including administration of dexmedetomidine at varying doses and time points (at the start of general anesthesia, as a bolus following extubation and as a CRI 15 min before the anticipated end of surgery) (23, 25, 26). All of these studies reported smoother extubation, lower incidence of post-anesthesia dysphoria and minimum effect on cardiovascular parameters. A recent study administered dexmedetomidine at a dosing regimen similar to the present study, determined that dexmedetomidine at 0.5 mcg/kg IV given as a slow bolus over 10 min to human patients after being administered fentanyl while under general anesthesia significantly improved post-operative agitation without prolonging recovery (10). The patients in that study had been discontinued from receiving inhalant anesthetic while our study dogs continued on inhalant during the study drug administration period. This could explain why the dogs in our study experienced a significantly prolonged recovery time. However, the improvement in recovery parallels the results of our study.

Administration of dexmedetomidine, an α_2_ adrenoreceptor agonist, results in an overall net increase in systemic vascular resistance (i.e. peripheral vasoconstriction), increasing arterial blood pressure. This increase in blood pressure potentiates the parasympathetic nervous system induced bradycardia. These cardiovascular effects may be significant (i.e. reductions in cardiac output); however, they are sometimes observed to a lesser extent with slow administration at a low dose in dogs (27, 28). In the present study, MAP increased by approximately 15-25% following the administration of 0.5 mcg/kg dexmedetomidine over 10 min when compared to SAL control. This is slightly higher than what was observed in previous canine studies (27, 28). Studies evaluating medetomidine at 1 mcg/kg administered over 10 min and dexmedetomidine at 0.5 mcg/kg administered over 6 min in dogs increased MAP by 19 and 13%, respectively, (27, 28).

In previously reported canine clinical trials, dexmedetomidine and medetomidine were administered following extubation to improve recovery quality from general anesthesia at 62.5 mcg/m^2^ (~ 3 mcg/kg) and 5 mcg/kg IV, respectively (8, 9). Dexmedetomidine administered IV at 62.5 mcg/m^2^ as a fast bolus post-extubation resulted in decreases in heart rate of almost 50% when compared to the saline control (8). In an experimental model in which isoflurane-anesthetized dogs received a fentanyl CRI at 5 mcg/kg/hr, the administration of dexmedetomidine at 2.5 mcg/kg IV during the recovery period as a fast bolus resulted in significant cardiovascular compromise (e.g. significant decreases in heart rate, cardiac index, and mixed venous oxygen tension) (29). Based on these findings, providing an alternative recovery dosing regimen for dexmedetomidine (i.e. lower dosing and administered at a slower rate prior to extubation) to mitigate these unwanted cardiovascular effects in healthy and especially cardiovascularly compromised patients is warranted. In the present study, heart rate decreased by about 10%, which is less of a change when compared to a previous study where heart rate decreased by 18% following the administration of 0.5 mcg/kg of dexmedetomidine over 6 min immediately followed by a 5 mcg/kg/hr dexmedetomidine CRI in anesthetized dogs. Despite the changes in HR observed in the previous study, cardiac index did not differ significantly when compared to baseline values in dogs (27). In a clinical study in dogs undergoing soft tissue or orthopedic surgery with an assigned ASA PS of II-IV, no clinically significant cardiovascular side effects were noted with the administration of a low-dose intraoperative dexmedetomidine CRI (25 mcg/m^2^ [~1 mcg/kg/hr]) (30). Similarly, anesthetized ASA PS I-II dogs undergoing soft tissue and orthopedic surgery administered a dexmedetomidine CRI at 1 mcg/kg/hr were reported to have maintained adequate overall tissue perfusion determined via perioperative serial arterial blood pressure and blood gas measurements (31). These previously mentioned and the present findings, in addition to further investigations, could lead to the utility of a slow low-dose bolus to assist in improving recovery following inhalant anesthesia in dogs with ASA PS > II. Contrary to these findings, another study reported a 15 and 22% reduction in cardiac index in dogs administered IV medetomidine at 2 and 5 mcg/kg, respectively, over 10 min (28). It is unclear whether these differences are significant amongst studies as differences in methodology (e.g. speed and dose of administration, clinical vs. research setting, and medetomidine vs. dexmedetomidine) could play a prominent factor. Regardless, mean arterial blood pressure and heart rate remained within clinically acceptable ranges.

Additionally, respiratory rate and arterial carbon concentrations have been previously reported to remain unaltered following the administration of dexmedetomidine at 0.5 and 3 mcg/kg/hr in isoflurane-anesthetized dogs (27). Similar to those findings, dexmedetomidine administered as in the present study also did not result in significant alterations in respiratory rate, however, carbon dioxide concentrations were not analyzed. A mild increase in respiratory rate did occur 15 min following the initiation of SAL treatment which may be associated with discontinuation of sevoflurane anesthesia 5 min prior to this measurement. The lack of significant change in RR in DEX at 15 min following treatment may be attributed to dexmedetomidine's ability to provide a more gradual and calming emergence from inhalant anesthesia. Additional studies evaluating the safety and efficacy of this dosing regimen in cardiopulmonary compromised patients is required.

There were several limitations to this study. The first limitation involves patient selection. Dogs selected to be enrolled were calm, cooperative, and did not require significant sedation to place an intravenous cannula. Patients that were perceived as nervous or aggressive during their pre-operative physical examination or that hydromorphone would not provide adequate sedation for IV cannulation were not included in the study by the principal investigator (SJ). It is difficult to determine if the prophylactic administration of dexmedetomidine using the present dosing protocol would produce similar results in aggressive or nervous dogs. Another limitation was that 2 (DEX *n* = 1; SAL *n* = 1) patients received IV fentanyl as their sole agent for premedication rather than hydromorphone. These 2 patients were already administered fentanyl CRIs prior to premedication and therefore an additional hydromorphone IM injection and the pain associated with it did not seem in the best interest of the patients as a clinical study. Despite the heterogenous population and the increased variability due to a non-uniform premedication protocol, a significant effect on NRS score was found in an appropriately powered study. Similarly, dogs received local regional blocks specific to the site of the surgical procedure. Due to the clinical nature of the study, the investigators believe it would be considered inappropriate to withhold these techniques for the sake of keeping consistency amongst experimental groups. However, the vast majority of dogs received the same local regional protocol and of similar frequency between DEX and SAL when compared to all other local regional protocols. Furthermore, duration of surgery and anesthesia were approximately 3.5 h and of shorter duration than the anticipated duration of analgesia provided by the local regional block. Therefore, it is unlikely that the local regional techniques performed had any impact on recovery quality in the present population. Another limitation is that the use of video recordings to score anesthetic recoveries has been reported to encourage researchers to exercise caution due to poor individual rater's agreement for specific scores (32). However, evaluating recovery overall was shown to maintain perfect agreement (32), which was similar to the present study as excellent agreement between reviewers was observed. Recovery quality was determined by assessing the 5 min following extubation. In humans, it has been previously reported that general anesthesia emergence delirium generally occurs within the first 10 min following extubation (2, 33). Furthermore, to allow for comparison amongst similar studies assessing recovery quality in anesthetized dogs receiving fentanyl CRIs, the authors opted to follow a similar duration of recovery assessment (i.e. immediately following extubation) (1, 21). Also, in the authors clinical experience, anesthesia recoveries of poor quality generally occur immediately following extubation and dogs rarely develop signs of dysphoria 5–10 min following a smooth extubation. However, it is difficult to determine at this time the effect of dexmedetomidine administered prior to extubation has on the later phases (>5 min post-extubation) of general anesthesia recovery in dogs. Finally, as a clinical study, several anesthetists and surgeons were involved in the perioperative management of these subjects. All individuals were briefed on study protocols prior to its initiation, however due to the clinical nature of the study freedom to manage the patients during the peri-operative period were granted as to not jeopardize patient-care. Despite the differences in anesthesia-care providers, physiological parameters and end-tidal sevoflurane concentrations remained consistent between groups. Ideally only one anesthetist would have managed all cases for consistence, however the use of various anesthetists in clinical trials has been reported (34).

In conclusion, the administration of low-dose dexmedetomidine IV as a slow bolus prior to discontinuing sevoflurane anesthesia can improve the recovery quality of healthy dogs administered a fentanyl CRI during general anesthesia undergoing elective or non-emergent orthopedic procedures. Although mild differences were noted in regards to duration of recovery, mean arterial blood pressure and heart rate, these changes are considered to have minimal clinical implications in healthy dogs. The patients enrolled in our study were healthy and undergoing elective or non-emergent orthopedic procedures. Future research is warranted to evaluate the application of this protocol to non-orthopedic or emergent procedures and in systemically compromised patients where the use of dexmedetomidine is not contraindicated.

## Data Availability Statement

The raw data supporting the conclusions of this article will be made available by the authors, without undue reservation.

## Ethics Statement

The animal study was reviewed and approved by Texas A&M University's College of Veterinary Medicine and Biomedical Sciences Institute's Animal Care and Use Committee and was approved (IACUC 2018-0045). Written and/or verbal consent, latter via phone, from the pet-owner was obtained for each subject. This method was approved by our institution.

## Informed Consent

The owners of all of the dogs included in the study were briefed on the study and consent was obtained before enrollment. Owners were also given a copy of the consent form for their records.

## Author Contributions

SJ: study design, data collection, and manuscript preparation. BS: study design, data collection, masked evaluation of recovery videos, and manuscript preparation. CB: data collection, masked evaluation of recovery videos, and manuscript preparation. SP: statistical analysis. JA: study design, statistical analysis, and manuscript preparation. All authors contributed to the article and approved the submitted version.

## Funding

Funding was obtained from a Texas A&M University GINN grant.

## Conflict of Interest

The authors declare that the research was conducted in the absence of any commercial or financial relationships that could be construed as a potential conflict of interest.

## Publisher's Note

All claims expressed in this article are solely those of the authors and do not necessarily represent those of their affiliated organizations, or those of the publisher, the editors and the reviewers. Any product that may be evaluated in this article, or claim that may be made by its manufacturer, is not guaranteed or endorsed by the publisher.
